# Circulating short chain fatty acids are associated with depression severity and predict remission from major depressive disorder

**DOI:** 10.1016/j.bbih.2025.101070

**Published:** 2025-07-19

**Authors:** Carmen Schiweck, Boushra Dalile, Alice Balliet, Mareike Aichholzer, Helena Reinken, Florian Erhardt, Julius Freiling, Aicha Bouzouina, Carmen Uckermark, Andreas Reif, Kristin Verbeke, Lukas van Oudenhove, Sharmili Edwin Thanarajah

**Affiliations:** a Goethe University Frankfurt, University Hospital, Department of Psychiatry, Psychosomatic Medicine and Psychotherapy, Germany; b Brain Research on Affective Mechanisms (BRAMLab), Laboratory of Biological Psychology, Research Unit Brain & Cognition, Faculty of Psychology and Educational Sciences, KU Leuven, Leuven, Belgium; c Laboratory for Brain-Gut Axis Studies (LabGAS), Translational Research Center in Gastrointestinal Disorders (TARGID), Department of Chronic Diseases, Metabolism, and Ageing, Faculty of Medicine, KU Leuven, Leuven, Belgium; d Laboratory of Digestion and Absorption (DigaLab), Translational Research Center in Gastrointestinal Disorders (TARGID), Department of Chronic Diseases, Metabolism, and Ageing, Faculty of Medicine, KU Leuven, Leuven, Belgium; e Fraunhofer Institute for Translational Medicine and Pharmacology, Frankfurt, Germany

## Abstract

**Background:**

Short-chain fatty acids (SCFAs) have been shown to improve depression-like behavior in animal models. However, their predictive value for depression outcomes in humans remains unclear. If SCFAs are predictive, this would provide strong justification for their use in diagnostic and intervention strategies. Therefore, the aim of our study was to determine whether baseline SCFA levels predict remission from depression over a six-month period. Secondary objectives included identifying the SCFA most strongly associated with depression outcomes and assessing the relationship between SCFA levels and inflammatory markers.

**Methods:**

A case-control study was conducted, including a pre-selected subset of 50 patients with major depressive disorder (MDD) at baseline, assessed at two time points (25 remitted, 25 non-remitted after six months), and 25 matched healthy controls (CTRL) from a convenience sample. The study was conducted at a university hospital, with fasting SCFA levels measured from high-quality blood samples. Depression severity was assessed using the Montgomery-Åsberg Depression Rating Scale (MADRS), and plasma concentrations of acetate, butyrate, and propionate were measured using gas chromatography-mass spectrometry at baseline and follow-up.

**Results:**

At baseline, plasma concentrations of propionate (t(71.2) = −2.38, P = 0.01, Cohen's d = 0.53) and butyrate (t(62.5) = −1.77, P = 0.041, Cohen's d = 0.41) were significantly lower in MDD participants compared to HC. Acetate levels did not differ significantly between groups (t(44.6) = −0.65, P = 0.26, Cohen's d = 0.16). Within the MDD group, baseline butyrate levels were associated with remission at follow-up (β = 2.79 ± 1.41, χ^2^(1) = 4.29, P = 0.038, OR = 16.3, 95 % CI: 1.01–262.4, AUC ROC = 0.67). Neither acetate (P = 0.43), propionate (P = 0.24), nor C-reactive protein (CRP) (P = 0.83) significantly predicted depression outcomes. Lasso regression identified butyrate as the primary predictor of depression severity at follow-up (β = 2.90 ± 1.39, χ^2^ = 4.38, P = 0.036).

**Conclusion:**

Patients with MDD exhibited lower baseline levels of butyrate and propionate compared to healthy controls. Higher baseline butyrate levels were associated with a greater likelihood of remission at follow-up. These findings suggest that butyrate may play a role in depression recovery, emphasizing the need for future studies to explore the therapeutic potential of butyrate-enhancing interventions in depression treatment.

## Highlights

• Plasma butyrate and propionate are lower in MDD and correlate with depression severity.
• C-Reactive Protein was not higher in MDD, but was associated with symptom severity.
• Higher plasma butyrate, but not propionate or acetate predicted remission from depression within 6 months.

## 1 Introduction

The role of gut microbial metabolites in pathological conditions, including psychiatric disorders like depression, is increasingly recognized. Among the most promising metabolites for potential applications in depression are short-chain fatty acids (SCFAs). SCFAs are volatile metabolites produced following bacterial fermentation of complex carbohydrates in the large intestine and are thought to mediate gut microbiota effects on brain and behaviour (Dalile et al., 2019; Liu et al., 2023). Previous experiments suggest that in rodents, oral administration of SCFAs in doses sufficient to raise blood concentrations show both antidepressant/anxiolytic and anti-inflammatory effects (Palepu et al., 2024; Sun et al., 2016). Nevertheless, research regarding their role in human MDD remains limited. Investigating SCFAs in humans is constrained by the inaccessibility of the colon, rapid splanchnic metabolism, and challenges in quantifying their low circulating concentrations (Boets et al., 2017). Most studies in MDD investigated fecal SCFAs, yielding inconsistent findings (Bruun et al., 2024), likely because fecal SCFAs represent only the non-absorbed fraction, ignoring *in situ* production, absorption, and molecular interactions (den Besten et al., 2013). In contrast, plasma SCFAs better capture the effects of interventions aimed to increase colonic SCFAs (Dalile et al., 2020, 2024). Recently, Liu et al. (2024) showed that plasma concentrations of the SCFAs propionate and butyrate - but not acetate - and their gut microbial producers, were lower in patients with major depressive disorder (MDD) compared to healthy controls. Fecal transplants from these patients induced inflammation and anxiety-like behaviors in mice, which were restored by a butyrate-enhancing probiotic (Liu et al., 2024).

Besides Liu et al., only two cross-sectional studies have investigated plasma SCFAs in depression, revealing lower acetate (Bot et al., 2020), but higher total SCFA concentrations in MDD patients (total concentrations also correlated positively with depression severity) (Liu et al., 2024; Yu et al., 2023); however, butyrate and propionate concentrations were not reported. The data by Liu et al. (2024) raise the question whether and which SCFAs could be useful to predict remission of depression after standard treatment. This prompted us to extend Liu et al.'s findings, by focusing on SCFA levels in relation to remission status over time. Specifically, we investigated whether circulating SCFAs a) differ between MDD patients and healthy controls, b) are associated with systemic inflammation and depression severity, and c) predict remission during treatment-as-usual. We predicted that 1) SCFA concentrations would be lower in patients with MDD compared to controls, 2) that C-Reactive Protein (CRP) would be higher in patients with MDD compared to controls and positively associated with symptom severity in MDD, 3) SCFA concentrations would be associated with inflammation (as measured by CRP) and that 4) higher SCFAs at baseline would predict remission from depression during treatment-as-usual.

## 2 Methods

The convenience sample selected for this study was part of a larger study with ethical approval, conducted according to the declaration of Helsinki (ENFORCE DRKS00029350, Ethical approval: 2021-508, and Genotyp Phänotyp: 425-14_8). Informed consent was obtained prior to all study related procedures. To assess the relevance of SCFA levels for treatment outcome, we selected patients based on remission status at follow-up based on a pre-defined score of Montgomery-Åsberg Depression Rating Scale (MADRS) ≤ 10 for remission or a MADRS >10 for non-remission. For the purpose of this manuscript, 25 patients with remitted status were available at the time of analysis. Therefore, we selected equal comparison groups, i.e., 25 matched healthy controls (HC) and 25 adult patients with MDD who did not remit at follow-up, but were comparable in terms of demographic data (age, sex and BMI). Their samples were sent to the laboratory based on this selection. Inclusion and exclusion criteria were: ≥18 years old, no pregnancy or breastfeeding, no type 1 diabetes, no severe neurological, rheumatological, or autoimmune disorders, no schizophrenia, bipolar disorder (manic episode), substance use disorder, or organic mental disorder. Patients with thyroid dysfunction on stable medication were included as long as lab results indicated adequate functioning. Given that the purpose of the study was to capture clinical reality, for inclusion, participants received care as prescribed by their psychiatrists. This often involved combinations of SSRIs, SNRIs, TCAs, and/or adjunct medications, no restriction was made here. Psychiatric diagnoses were assessed (for MDD patients) or excluded (for healthy controls) by a trained interviewer using the Mini International Neuropsychiatric Interview (MINI) diagnostic interview, and symptom severity was assessed using the MADRS interview.

### 2.1 Procedure

All participants were invited to the clinic and gave written informed consent. The screening visit, including the MADRS and MINI interviews, was performed. If the screening criteria were fulfilled, participants were included. A blood draw by venipuncture was performed between 7.00h and 10.00h in the morning, after an overnight fast for both the baseline (control and MDD groups) and follow-up (MDD group only) visits. After the blood draw, questionnaires and computer tasks were performed (not relevant for the present analysis). The follow-up assessment was conducted either upon remission as assessed by the MADRS interview or, if remission was not achieved, after a maximum of 6 months.

### 2.2 C-reactive protein and SCFA analysis

Serum CRP was measured in fasted morning samples via standard hospital laboratory protocols. For SCFA analysis, plasma was collected using K3-EDTA tubes (S-Monovette®, Sarstedt, 9 mL), immediately placed on ice, centrifuged (4 °C, 2000g, 10 min), and frozen at −70 °C within 30 min (stored between 2021 and 2024). The circulating SCFAs were later quantified in plasma samples using gas chromatography-mass spectrometry after derivatization with 2,4-difluoroaniline and ethyl acetate extraction (Vandermeulen et al., 2024).

### 2.3 Statistical analysis

A sensitivity power analysis including n = 25 CTRL versus n = 50 MDD yielded 80 % power to detect a medium effect size (Cohen's d = 0.61) based on a one-tailed *t*-test (given the directional hypothesis). Demographic characteristics were compared between the three groups using the Kruskal Wallis test and, if significant, were followed up by Dunn's test. Variables were transformed to satisfy assumptions for statistical testing. Robust Analysis of Variance (rANOVAs) was used to compare group differences in SCFA concentrations at baseline. Robust regressions were performed to assess the association between MADRS scores, CRP and SCFA concentrations. Partial Least Squares (PLS) were performed for predictive analyses. To this end, baseline propionate, butyrate, and CRP were entered as the independent variables in PLS regression with baseline or follow-up MADRS score as the sole dependent variable for baseline analyses and for prediction of follow-up analyses, respectively. Details on the factor solutions can be found in the Supplementary Material.

## 3 Results

**Fig. 1:**
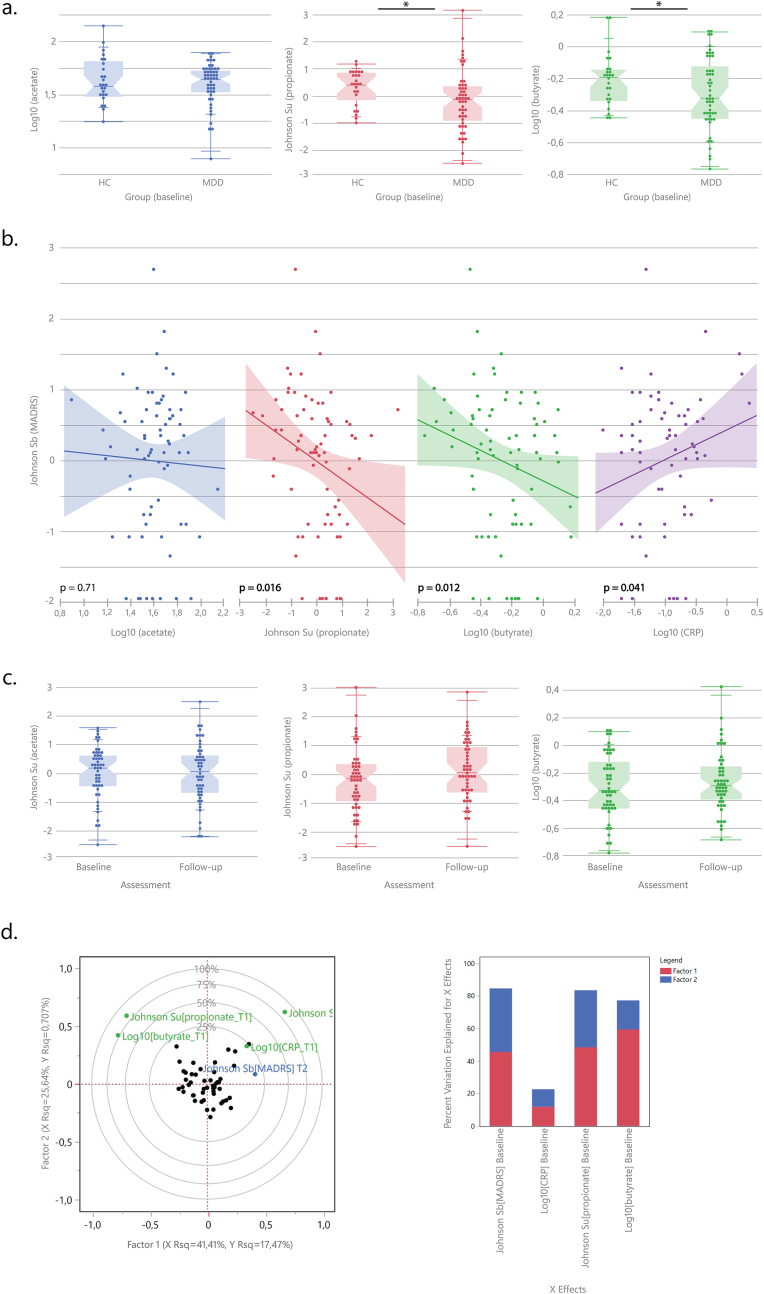
Plasma SCFA concentrations in HC and MDD patients at baseline **(a).** Associations between MADRS scores and SCFAs at baseline **(b).** Plasma SCFA concentrations at baseline and follow-up in MDD patients **(c)**. Partial Least Squares (PLS) (left) correlation loading plot for the model explain MADRS score at follow-up in MDD patients (green dots are independent (X) variables, blue dot is the dependent (Y) variable, black dots are individual patients); (right) plot showing the percentage variation for each independent (X) variable explained by the two factors **(d)**. Abbreviations - HC: Healthy controls, MDD: Major Depressive Disorder, MADRS: Montgomery and Asberg Depression Rating Scale. (For interpretation of the references to colour in this figure legend, the reader is referred to the Web version of this article.)

Demographic data and antidepressant medication regiment can be found in Table S1 and S2. Plasma propionate [t(71.2) = -2,38, p = 0.01, Cohen's d = 0.53] and butyrate [t(62.5) = -1.77, p = 0.041, d = 0.41], but not acetate [t(44.6) = -0.65, p = 0.26, d = 0.16] concentrations were lower in participants with MDD compared to HC [Fig. 1A] and were negatively associated with depression severity at baseline across the entire sample (robust regression on MADRS score, propionate p = 0.016, butyrate: p = 0.012, acetate, p = 0.71) [Fig. 1B]. CRP was not different between groups [t(54.9) = 1.14, p = 0.13, d = 0.27], and was not associated with propionate (p = 0.65) or butyrate (p = 0.13) but was positively associated with symptom severity (p = 0.041) [Fig. 1B] and negatively with acetate (p = 0.02) across the entire sample.

Partial Least Squares (PLS) regression with baseline propionate, butyrate, and CRP concentrations as independent variables confirmed the importance of all three variables in explaining baseline depression severity (two-factor solution explaining 9.7 % of the variance, variable importance for projection (VIP) threshold of all>0.8) (details in supplement). SCFA concentrations were stable over time and did not change from baseline to follow-up within the MDD group (acetate [F(1,49) = 1.10, p = 0.30], propionate [F(1,49) = 2.31, p = 0.13], butyrate [F(1,49) = 1.27, p = 0.27] [Fig. 1C].

Assessing the predictive value of plasma SCFAs for remission status (i.e., remitted vs. non-remitted) at follow-up revealed significant predictive effects for baseline butyrate (β = 2.79 ± 1.41, χ^2^(Dalile et al., 2019) = 4.29, p = 0.038, OR 16.3 [1.01–262.4, AUC ROC curve = 0.67], but not acetate (p = 0.43), propionate (p = 0.24), or CRP (p = 0.83). The importance of butyrate was confirmed in a logistic regression with LASSO penalization and leave-one-out cross-validation showing that butyrate emerged as the sole significant predictor of remission status post-treatment (β = 2.90 ± 1.39, χ^2^ = 4.38, p = 0.036), with the coefficients for propionate and CRP being shrunk to zero by the LASSO procedure (for full results, **see supplements**). Similar results were observed for depression severity as a continuous variable at follow-up: PLS regression with baseline propionate, butyrate, and CRP concentrations and baseline depression severity as the independent variables explained 18.2 % of the variance in follow-up depression severity (two-factor solution), with baseline depression severity, propionate, and butyrate, but not CRP, exceeding the VIP threshold of 0.8 (**details in supplement**), indicating that a combination of these variables may be useful in explaining future depression severity [Fig. 1D]

## 4 Discussion

In the present study we found lower circulating propionate and butyrate concentrations in patients with MDD compared to HCs, supporting the notion of diminished SCFAs and potentially, reduced bacterial SCFA-producers in depression (Valles-Colomer et al., 2019). Importantly, our longitudinal data also provides first human evidence that butyrate is associated with lower depression severity and may predict remission during treatment-as-usual.

Our findings of lower butyrate and propionate concentrations in MDD at baseline compared to the healthy controls is in line with the report by Liu et al. (2024) and corroborates the usefulness of assessing SCFA concentrations in plasma. The novel finding, showing that particularly butyrate is associated with remission from depression during a 6-month window, raises the question regarding the mechanisms that could be implicated in this association. One possible explanation comes from previous preclinical studies which found that SCFAs augment the effect of anti-depressants (Liu et al., 2023; Klünemann et al., 2021; Schroeder et al., 2007). Specifically, Schroeder et al. found that both chronic and acute butyrate administration in combination with the selective serotonin reuptake inhibitor (SSRI) fluoxetine in normal mice outperformed administration of fluoxetine alone on antidepressive-like effects on behavioural tests (Schroeder et al., 2007). These findings provide an intriguing possible explanation for the observed effects in our sample who received treatment as usual.

A second possible explanation is that SCFA exert an anti-depressant effect through their anti-inflammatory capacity. It is well known that chronic low-grade inflammation plays a role in a subset of patients with depression (Liu et al., 2024; Osimo et al., 2020). In preclinical studies, prolonged administration of an SCFA mixture increased plasma and faecal SCFAs, decreased proinflammatory cytokines, and exhibited antidepressant and anxiolytic effects in a model of treatment-resistant depression in rats (Palepu et al., 2024). Similarly, administration of butyrate alone in mice counteracted endotoxin-induced depressive behaviour and neuroinflammation (Qiu et al., 2020). Reducing inflammation through SCFA could thus indirectly improve depression severity. Indeed, this mechanism has been suggested as the driving force behind the results of Liu et al. (2024). However, while in their sample, the authors highlighted the inflammatory pathways driven by microbial dysbiosis and the inflammatory depression subtype (as assessed by a CRP level above 1.11 mg/L), we did not find such strong effects of inflammation in the current analysis. Instead, our results suggest that the relevance of butyrate extends beyond inflammatory subtypes of MDD. That is, despite no differences in CRP between the groups and lack of association between butyrate concentrations and CRP levels, butyrate was still independently linked to depression severity. While this does not exclude inflammation as a contributing mechanism, it implicates additional mechanisms such as direct effects on the blood-brain barrier, neuroinflammation, neurotransmitter synthesis, and neuroplasticity via BDNF (Dalile et al., 2019; Liu et al., 2023). Indeed, butyrate administration reversed depressive behaviour in a model of chronic unpredictable mild stress partially due to increased 5-HT concentration, increased BDNF expression, and ameliorating blood–brain barrier (BBB) impairments (Sun et al., 2016).

Another question logically arising from our data, is whether intervention studies with butyrate can show anti-depressant effects in humans. Several animal studies exist, which indeed show beneficial effects of SCFA administration (Palepu et al., 2024; Sun et al., 2016; Liu et al., 2024), but to the best of our knowledge, no such study has been conducted in humans with depression. Our previous work in healthy male participants showed no effects of colonic administration of a SCFA mixture (Dalile et al., 2020) nor butyrate alone (Dalile et al., 2024) on depression scores administration, yet the former results in reduced cortisol reactivity to acute stress. Intervention studies using SCFAs and specifically butyrate-enhancing bacterial strains or prebiotic substrates are thus urgently required to shed light on the beneficial effects of SCFAs and the mechanisms driving these effects.

An important caveat of the present study is the inability to definitively localize the site of butyrate's action. Our prior work demonstrated that colonic administration of known quantities of SCFAs leads to increased systemic uptake, albeit with substantial interindividual variability. Notably, this variability correlates with differences in the magnitude of psychobiological responses (Dalile et al., 2020). These findings suggest that butyrate may act through multiple pathways - locally in the gut via interaction with free fatty acid receptors (FFARs), and systemically via absorption into the circulation and interaction with gut-brain communication mechanisms. To clarify these potential routes of action, future studies should compare SCFA administration in the upper gastrointestinal tract - where systemic absorption is known to be higher - with colonic delivery, which results in relatively lower systemic availability due to utilization of butyrate by colonocytes. This approach may help determine which route is more effective in modulating psychobiological outcomes of interest, including depression scores. While lower plasma butyrate levels indicate changes in the gut-microbiome composition with depletion of butyrate producing taxa, microbiome data from this cohort were not available to investigate this association. Similarly, here, only a subset of participants had dietary data available. Future studies should include dietary habits -particularly fiber intake - as potential confounders. Lastly, this study represents a small dataset including 50 MDD patients and 25 healthy controls. Findings should be interpreted cautiously until replication is possible in larger samples.

To conclude, by integrating a longitudinal therapeutic perspective, our study adds novel human data which provides new evidence that SCFAs, and in particular butyrate, may play an important role in the trajectory of MDD. These insights emphasize the potential of butyrate as a therapeutic target and encourage exploring its mechanisms across diverse subtypes of MDD.

## CRediT authorship contribution statement

**Carmen Schiweck:** Conceptualization, Data curation, Formal analysis, Investigation, Methodology, Project administration, Writing – original draft, Writing – review & editing. **Boushra Dalile:** Investigation, Methodology, Writing – original draft, Writing – review & editing. **Alice Balliet:** Data curation, Investigation, Writing – original draft, Writing – review & editing. **Mareike Aichholzer:** Data curation, Writing – review & editing. **Helena Reinken:** Data curation, Writing – review & editing. **Florian Erhardt:** Data curation, Writing – review & editing. **Julius Freiling:** Data curation, Writing – review & editing. **Aicha Bouzouina:** Data curation, Writing – review & editing. **Carmen Uckermark:** Data curation, Writing – review & editing. **Andreas Reif:** Resources, Supervision, Writing – review & editing. **Kristin Verbeke:** Conceptualization, Formal analysis, Methodology, Writing – original draft, Writing – review & editing. **Lukas van Oudenhove:** Conceptualization, Formal analysis, Investigation, Methodology, Visualization, Writing – original draft, Writing – review & editing. **Sharmili Edwin Thanarajah:** Project administration, Supervision, Validation, Writing – original draft, Writing – review & editing.

## Declaration of competing interest

AR has received honoraria for lectures and/or advisory boards from Janssen, Boehringer Ingelheim, Compass, GH Research, SAGE/Biogen, LivaNova, Medice, Shire/Takeda, Newron, MSD, AbbVie and cyclerion and has received research grants from Medice and Janssen. MA received honoraria for an advisory board of Janssen Cilag. received personal fees for a scientific advisory board by Janssen. All other authors have no competing interests to declare.

## Data Availability

Data will be made available upon request.
