# Supplementary material for: Circulating short chain fatty acids are associated with depression severity and predict remission from major depressive disorder
Source: Brain Behav Immun Health. 2025 Jul 19;48:101070. doi: 10.1016/j.bbih.2025.101070 (PMC12320157; doi:10.1016/j.bbih.2025.101070)
Supplement: Multimedia component 1 [file mmc1.docx]

**Supplementary Material**

| **Table S1: Demographic characteristics between groups. CRP: C-Reactive Protein; na-not applicable.** | | | | | | |
| --- | --- | --- | --- | --- | --- | --- |
| **Group** | **Age (Mean ± SD)** | **BMI (Mean ± SD)** | **Sex (Male/ Female)** | **MADRS Median (range)** | **CRP Median (range)** | **Smoker (n, %)** |
| **HC** | 37.32 ± 13.49 | 25.98 ± 4.47 | 9/16 | 2 (0-8) | 0.08 (0.02-0.57) | 5 (33%) |
| **MDD** | 39.76 ± 15.17 | 25.09 ± 4.51 | 10/15 | 31 (18-49) | 0.1 (0.01-1.78) | 17 (71%) |
| **rMDD** | 40.2 ± 15.53 | 26.77 ± 6.88 | 10/15 | 24 (11-47) | 0.12 (0.02-2.34) | 11 (44%) |
| **Kurskal-Wallis Test (3 groups)** | X2=0.351, p=0.839 | X2=0.572, p=0.751 | X2= 0.11244, p=0.945 | X2=50.52, p<0.001 | X2=1.17, p=0.557 | X2=6.14, p=0.046 |
| **Post-hoc Dunn’s test** | na | na | na | HC< MDD:p<0.001  HC< rMDD:p<0.001  MDD = rMDD:p=0.120 | na | HC<MDD: p=0.049  HC=rMDD:p=0.739  MDD=rMDD:p=0.108 |

**Table S2: Antidepressant medication of MDD groups split by remission status at T2.** Comparison of proportions revealed no significant difference: X2 =6.72, df = 5, p-value = 0.151 SSRI: Selective Serotonin Reuptake Inhibitors, SNRI: Serotonin-Norepinephrine Reuptake Inhibitor, TCA: Tricyclic Antidepressant, TeCA: Tetracyclic Antidepressant.

| **Treatment Group** | **MDD (n=25)** | **rMDD (n=25)** |
| --- | --- | --- |
| SSRI monotherapy | 4 (16%) | 9 (36%) |
| SNRI/NDRI monotherapy | 7 (28%) | 2 (8%) |
| SSRI or SNRI + atypical Antipsychotic / Other | 8 (32%) | 7 (28%) |
| TCA/TeCA (+Others) | 4 (16%) | 2 (8%) |
| Unmedicated | 2 (8%) | 5 (20%) |
| Missing | 0 (0%) | 0(0%) |

**Partial Least Square Regression Information**

*Baseline depression severity*

Baseline propionate, butyrate, and CRP were entered as the independent variables in PLS regression with baseline MADRS score as the sole dependent variable. A solution with two X factors cumulatively explained 83.6 % of the variance in X (independent variables) and 9.7% of the variance in Y (baseline MADRS score, dependent variable). All three independent variables exceeded the VIP threshold of 0.8 (propionate: 1.04, CRP: 1.00, butyrate: 0.95). The first factor, cumulatively explaining 51.3% of the variance in X and 9.07% of the variance in Y, had negative loadings for propionate (-0.63) and butyrate (-0.55), explaining 59.4 and 67% of their variance, respectively, and a positive loading for CRP (0.42), explaining 27.4% of its variance. The second factor cumulatively explained 32.6 of the variance in X and 0.6% of the variance in Y, with positive loadings for CRP (0.81) and, to a lesser extent, butyrate (0.47) and propionate (0.34), explaining 64.9%, 21.9%, and 11.1% of their variance, respectively

*Follow-up depression severity*

Baseline propionate, butyrate, CRP, and MADRS score were entered as the independent variables in PLS regression with follow-up MADRS score as the sole dependent variable. A solution with two X factors cumulatively explained 67% of the variance in X (independent variables) and 18.2% of the variance in Y (follow-up MADRS score, dependent variable). Baseline MADRS score, butyrate, and propionate, but not CRP, exceeded the VIP threshold of 0.8 (1.27, 1.09, 0.91, and 0.61, respectively). The first factor, cumulatively explained 41.4 of the variance in X and 17.6% of the variance in Y. It had negative loadings for butyrate (-0.59) and propionate (-0.54), explaining 59.5 and 48.5 % of their variance, respectively, and positive loadings for baseline MADRS score (0.53) and CRP (0.27), explaining 45.7 and 12.0% of their variance, respectively. The second factor cumulatively explained 25.6% of the variance in X and 0.7% of the variance in Y. It had positive loadings for baseline MADRS score (0.62), propionate (0.58), butyrate (0.42) and CRP (0.32), explaining 39.0, 35.0, 17.8, and 10.7 % of their variance, respectively.
